# The increase in total knee replacement surgery in Taiwan: A 15-year retrospective study

**DOI:** 10.1097/MD.0000000000011749

**Published:** 2018-08-03

**Authors:** Fu-Huang Lin, Hsiang-Cheng Chen, Chin Lin, Yu-Lung Chiu, Herng-Sheng Lee, Hung Chang, Guo-Shu Huang, Hsueh-Lu Chang, Shih-Jen Yeh, Wen Su, Chih-Chien Wang, Sui-Lung Su

**Affiliations:** a School of Public Health; b Division of Rheumatology/Immunology/Allergy, Department of Internal Medicine, Tri-Service General Hospital, National Defense Medical Center, Taipei; c Department of Pathology and Laboratory Medicine, Kaohsiung Veterans General Hospital, Kaohsiung; d Department of Physiology and Biophysics; e Division of Thoracic Surgery; f Department of Radiology, Tri-Service General Hospital, National Defense Medical Center, Taipei; g Department of Research and Development, Da-Yeh University, Changhua; h Department of Nursing; i Department of Orthopedics, Tri-Service General Hospital, National Defense Medical Center, Taipei, Taiwan, ROC.

**Keywords:** database, medical resources, total knee replacement

## Abstract

Total knee replacement (TKR) is considered as one of the most success among clinical interventions for patients with who suffering from knee osteoarthritis (OA). We sought to estimate the incidence of TKR using demographics, incidence rates, lengths of hospital stay, and costs from 1996 to 2010 by analyzing Taiwan's National Health Insurance Research Database. A total of 154,553 patients obtained primary TKR surgery between 1996 and 2010. The diagnosis code for knee OA and the procedure code for TKR were selected from the records. To compare the rate of TKR between covariables, we calculated the TKR risk ratios and 95% confidence interval (CI) of these variables (gender, age, age group, and primary diagnoses). A 2-tailed *P*-value of .05 was considered statistically significant. The statistical package SPSS version 20.0 (SPSS, Chicago, IL) was used to conduct all the statistical analyzes. We analyzed 154,553 TKRs performed by surgeons in Taiwan from 1996 to 2010. The overall crude incidence increased from 26.4 to 74.55 TKR per 100,000 inhabitants from 1996 to 2010. TKR incidence for the 70 to 79 years age group increased from 227 to 505 per 100,000 people from 1996 to 2010. The age-standardized rate ratios for TKR of women to men ranged from 2.5 to 3.0. The mean average length of stay in hospital was 15 days in 1996 and decreased to 8 days in 2010. During the study period, the adjusted mean cost per patient decreased from US$7485 to US$4827. Health expenditures for TKR were 5% of total National Health Insurance expenditure every year. Over the 15-year period, Taiwan's TKR incidence tripled, which is consistent with population ageing. Arthritis will be a major public health issue in the ageing population in the future.

## 1 Introduction

Osteoarthritis (OA) is a degenerative joint disease that causes progressive disorder of joint function. It is the leading cause of disability and poor quality of life among the elderly in developed countries.^[1]^ Knee OA is also prevalent in East Asian countries.^[2]^ The occurrence of knee OA increases with age, particularly in women. In adults over the age of 45, 6% to 13% of men are affected, whereas 7% to 19% of women are affected, demonstrating a 45% lower risk of incidence for men.^[3,4]^ The prevalence of OA is markedly higher among women than men and increases noticeably with age.^[5,6]^ Knee OA occurs in 12% of American adults 65 years old or elder, and in 13% of women and 10% of men 60 years old or older in the United States.^[7–9]^ The prevalence of OA is 15% in women and 5.6% in men 60 years old or older in Beijing, China.^[10]^ The current occurrences of OA among the elderly population in Taiwan is about 37% in individuals over 50 years old.^[11]^ The demographic crisis of ageing is spreading worldwide, including in Taiwan. Taiwan has been defined as an ageing society (according to the United Nation classification) since 1993, when the percentage of the population over 65 years old reached 7%. The percentage of the Taiwan population that is over 65 years old is forecast to surpass 14% by 2017, rapidly increasing to 20% by 2025.^[12–14]^

Total knee replacement (TKR) is a general surgical operation of high success rate that improves the function and quality of life in patients with disorder in the knee joint.^[15,16]^ The rate of TKR has been steadily increasing over the last 2 decades.^[17,18]^ This trend is also occurring in Taiwan, but a full view of the epidemiology has not been obtained.

Up to 96% of all Taiwan residents are enrolled in the National Health Insurance (NHI) program; this high rate of enrolment extends as least as far back as 1996.^[19]^

In Taiwan, few medical reports have investigated the trends in the prevalence of primary TKRs, and information from the NHI database is considered appropriate for assessment of epidemiologic features of TKR in Taiwan. We used the NHI research database to investigate the epidemiologic features of TKR in Taiwan. We sought to estimate the incidence of TKR from 1996 to 2010 by demographics, incidence rates, lengths of stay, and medical expenses.

## 2 Materials and methods

The information retrieved from the National Health Insurance Research Database in Taiwan was fully representative of all population groups, as over 99% of the population of Taiwan is included in the government-run health insurance program (National Health Research Institutes, ROC, 2011).

We analyzed data from Taiwan's National Health Insurance (NHI) from 1996 to 2010 released by the National Health Research Institutes for public research purposes. We used the administrative claims data from the NHI form 1996 to 2010 for our analysis. In this study, we analyzed data for all patients who received a primary TKR procedure (ICD-9-CM) procedure code 81.54 from 1996 to 2010. All TKR procedures performed for the treatment of chronic or complicated diseases and traffic accidents were excluded from the analysis. We analyzed 154,453 TKR procedures.

Research variables in this study also included sex (male and female), age (5 groups: <50 years old, 50–59 years old, 60–69 years old, 70–79 years old, and ≥80 years old) and primary diagnoses (4 groups categorized as: OA, rheumatoid arthritis [RA], avascular necrosis [AVN], and other; the ICD-9-CM codes for primary diagnoses of OA, RA, and AVN were 715.00 to 715.98; 714 and 714.0; and 733.40, 733.42, and 733.49 and other ICD-9CM codes, respectively), cost, average length of stay (ALOS), total charge (million USDs), and expenditure rates of NHI (permillage).

To reflect real dollar values, all dollar values at the end of each year were adjusted to 2011 Taiwan currency values. All hospital charges were then converted from Taiwan dollars to US dollars using an exchange rate of 30:1, based on the average exchange rate over the 1996 to 2010 period. For factoring of inflation, the price index has a base year of 2010 (Directorate-General of Budget, Accounting and Statistics, Executive Yuan, ROC [Taiwan]), previous prices are being compared to prices in that time period. The influences of population characteristics, characteristics of places of care, disease patterns of medical resource utilization (in-hospital medical cost and length of hospital stay), and quality of care (in-hospital mortality and postoperative infections) are described by the number of cases or means with standard deviation. To compare the rates of TKR between covariables, we calculated TKR risk ratios and 95% CIs of these variables (gender, age, age group, and primary diagnoses). Statistical analyzes were conducted using SPSS version 20.0 (SPSS, Chicago, IL). All tests were 2-sided, and *P-*values of <.05 were considered statistically significant.

## 3 Results

### 3.1 Demographic trends

Baseline clinical characteristics of participants with TKR are shown in Table 1. In all, 154,553 TKR surgeries were performed in Taiwan during the 15-year study period from 1996 to 2010.

**Table 1:**
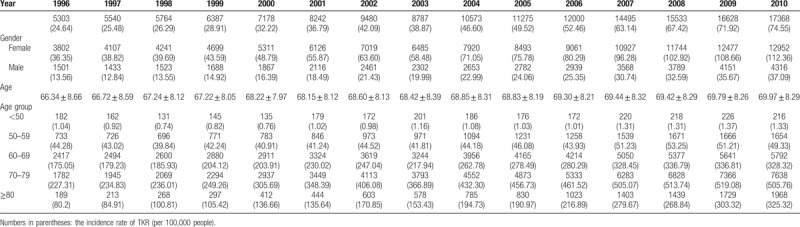
Number of cases of total knee replacement (TKR) from 1996 to 2010.

The number of TKRs increased from 5303 (24.64 per 100,000) in 1996 to 17,368 (74.55 per 100,000) in 2010 (an increase of 202.56%). An increasing trend was found in the TKR rate in both sexes. In 1996, the rate of TKR in males was 13.56 per 100,000 persons, which increased to 37.09 per 100,000 persons over the years 1996 to 2010 and in females the rate was 36.35 per 100,000 persons, which increased to 112.36 per 100,000 persons in 2010 (an increase of 173.53% and 209.11%, respectively). The rate of incidence of TKR in females was approximately 2.5 to 3 times that of males from 1996 to 2010. The mean age was 68.83 years in all individuals, and the average age of patients receiving TKR increased from 1996 (66.34 years old) to 2010 (69.97 years old). During the study period, the average age of TKR patients gradually increased by 3.63 years. The rate for TKR increased for all age groups from 1996, and the increase was most obvious among older adults, reaching a peak over 70 to 79 years old.

**Figure 1:**
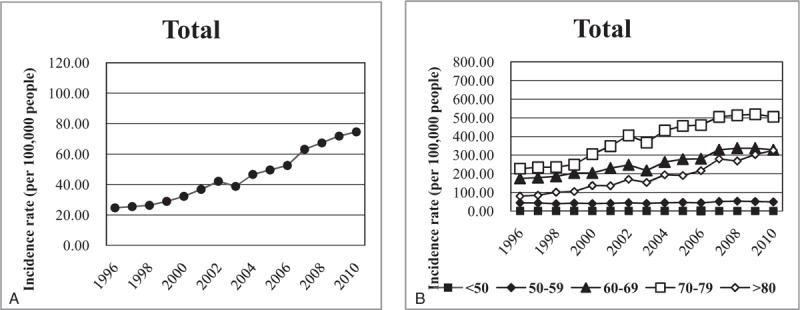
Incidence rates of primary total knee replacements from 1996 to 2010. The rates were calculated as register count per 100,000 persons in the population defined by the National Health Insurance. (A) Crude incidence rates. (B) Incidence rates stratified by age.

**Figure 2:**
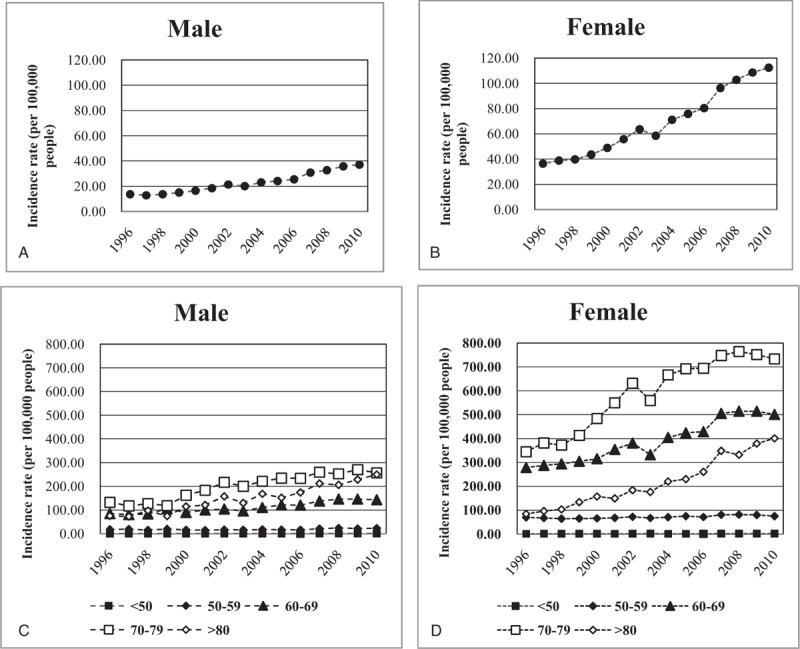
Incidence rates of primary total knee replacements from 1996 to 2010 stratified by gender. The rates were calculated as the register count per 100,000 persons in the population defined by the National Health Insurance. (A) Crude incidence rates in men. (B) Crude incidence rates in women. (C) Incidence rates stratified by age in men. (D) Incidence rates stratified by age in women.

The rate of TKR tripled, from 24.64 to 74.55 per 100,000 between 1996 and 2010. The distribution of age in TKR is shown in Figure 1, where the highest incidence age group was 70 to 79 years old. However, there was a large increase in the distribution in TKR in the ≥80 age group, whose incidence rate increased approximately 4.1 times. The incidence rates of TKR stratified by sex are also shown in Figure 2. The incidence rates increased over the 14 years of the study and were much higher in women than in men. The incidence rates of TKR in men increased 2.7 times, from 13.56 to 37.09 per 100,000, and incidence rates in women increased 3.1 times, from 36.35 to 112.36 per 100,000. The 70 to 79 age group had the highest increase of TKR for both men and women. There was also a severe increase in TKR among women in the ≥80 age group, where the incidence rate increased approximately 4 times. However, there seemed to be a similar incidence rate of TKR in the 70 to 79 and ≥80 age group in men.

### 3.2 Trends in medical resources

Table 2 presents the changing trends in basic characteristics and medical resources allocated to TKR. The gender percentages also show a similar result to the incidence rate of TKR for females compared with males (71.70–75.00% vs 25.00–28.30%) in 1996 to 2010. The principal diagnosis during the study period was OA, which ranged from 90.31% to 96.94%. The average cost to individuals undergoing TKR declined from $7485.32 to $4826.61 (a decrease of 35.52%). However, total charges increased from $39.69 million to $83.35 million (an increase of 110.00%). The rate of expenditure by the NHI for TKR increased slightly (4.64 to 5.00 permillage in 1996–2010, an increase of 7.76%). In addition, the ALOS for TKR notably declined. ALOS decreased from 15.44 to 8.19 days (a decrease of 46.96%).

**Table 2:**
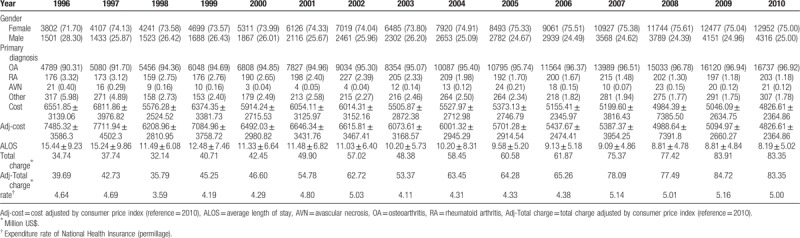
Distribution of medical resource usage in total knee replacement from 1996 to 2010.

### 3.3 Predictors for TKR

The rate ratios of the risk factors for TKR are shown in Table 3. The rate ratio (RR) of TKR per year was 1.09 (95% CI: 1.08–1.09); for females compared with males, it was 1.66 (95% CI: 1.63–1.69). In comparison with the <50 age group, the RR for TKR was 5.45 (95% CI: 5.16–5.75) in the 50 to 59 age group, 15.21 (95% CI: 14.45–16.00) in the 60 to 69 age group, 21.23 (95% CI: 20.17–22.34) in the 70 to 79 age group and 16.24 (95% CI: 15.30–17.25) the in ≥80 age group. Patients with RA and AVN in their primary diagnoses had a lower risk for TKR (RR = 0.15, 95% CI: 15.30–17.25 and RR = 0.007, 95% CI: 15.30–17.25, respectively) than those with OA.

**Table 3:**
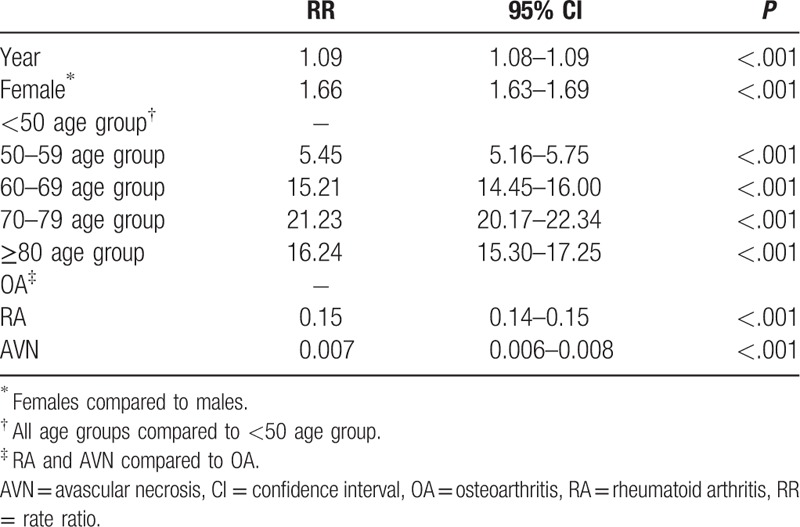
Rate ratio of covariables for primary total knee replacement rate.

## 4 Discussion

Our study found that the rate of TKR increased over the years 1996 to 2010 and the increase was much larger when women compared with men. In 1996, the rate of primary TKR was 24.64 per 100,000 persons, which gradually increased to 74.55 per 100,000 persons in 2010. This is an increase of 202.56% over 15 years. This increase in the rate is in agreement with the results of studies conducted by Tien et al and Kumar et al.^[20,21]^

According to Tien's study, the prevalence of TKR was 22.86 per 100,000 persons in 1996 and it increased to 54.95 per 100,000 persons in 2004, which is an increase in rate of 140.38%.

Kumar's study showed that the prevalence rate of TKR was 28.5 per 100,000 persons in 1998 and increased to 56.8 per 100,000 persons in 2009, which in an increase in rate of 99.1%.^[20,21]^

The age-adjusted TKR rate increased by 81.5% (from 162 to 294 per 100,000) in Wisconsin, in the United States from 1990 through 2000.^[17]^ Primary TKR increased from 6.3 per 10,000 in 1995 to 11.0 per 10,000 in 2004 in Southern California, which is an increase rate of 74.60%.^[22]^ Ravi's study used the databases of the Healthcare Cost and Utilization Project and the Institute for Clinical Evaluative Sciences to estimate the prevalence of TJA of hip and knee in the United States and Ontario, Canada, respectively. The increase in rates of TKA from 2001 to 2007 was 59% in the United States and 73% in Ontario.^[23]^ The annual primary TKA volume increased 161.5% from 93,230 to 226,177 between 1991 and 2010.^[24]^ There were 10,132 primary TKA for OA in Australia in 1994, which increased by 42.8% to 14,472 in 1998.^[25]^ Primary TKA rates increased by 407% in Korea from 2001 to 2010, according to data from the Health Insurance Review and Assessment Service of Korea.^[26]^ Taiwan became an ageing society in 1993, when the proportion of those aged over 65 exceeded 7% of the country's population.^[12]^ The proportion of people over 65 years old was 9% to 10.7% between 2002 and 2010.^[27]^ Policies for an ageing population should include health care utilization of TKR and prevention of OA. This seems to have increased the annual incidence rate of TKR between 1996 and 2010. However, there was a downward trend in incidence of TKR in 2003. This may be attributed to the pandemic of severe acute respiratory syndrome (SARS) in that year. In that year, many people were afraid of getting SARS, and avoided going to hospitals.^[28]^

The TKR differed between the genders. The TKR rate was higher in women. Similar results for TKR studies with relation to gender have been reported in other countries. Many studies have reported that knee OA is more common in women than in men.^[29–32]^ Some studies have also shown that TKR rates are higher in women than in men. In Sweden, the TKR rate is 1.93 times greater in women than in men, and the gender discrepancy in TKR rate of a similar order of magnitude to Sweden was also found in the United States.^[17,33]^ Our study shows that the rate of TKR was 2.5 to 3 times larger in women than in men in Taiwan, slightly greater than that of previous studies.^[20,21]^

The rates of TKR presented a rising tendency with increases in patient age. The study results resemble other studies in Western countries and the Asian area, where TKR rates were high in the 70 to 79 age group. The increment in the rate of symptomatic OA with the escalation in age demonstrates this tendency.^[1,21,34]^ The mean age of patients of TKR has risen. Advances in the medical management of OA may mean patients postpone joint replacement surgery, leading to an incremental rise in the mean age of patients receiving TKR.^[21]^

In the United States, the main diagnosis of TKR patients is OA (approximately 86–87%).^[18,35]^ A study by Tien et al in Taiwan noted that about 94% of TKR patients had OA in 2002 to 2004.^[20]^ Our study showed that 96.9% of TKR patients had OA in 2010. It also seems that the incidence rate of TKR has increased annually. There is a close association between the ageing society and the risk of OA.

As far as we know, there has been no epidemiologic study investigating TKR utilization in the Taiwanese population over a long period. To control the dramatic growth in medical expenditures, the administration of NHI is gradually changing its payment system from a fee-for-service payment to case payment.^[36]^ According to the data from Taiwan's Health Insurance Bureau, about 6500 people undergo TKR every year. TKR is a surgical operation that has high utilization and high consumption of medical resources in Taiwan.^[37]^ We found that the trends in the mean cost per patient and the ALOS for TKR have fallen slowly from 1996 to 2010. There was a small peak in the cost and the mean length of stay for TKR in 1999. The trend analysis of medical resource utilization showed that the mean cost per patient and ALOS markedly declined; the reduction may have been due to advances in medical technology and sciences.^[38]^ In the United States, LOS reduced from 7.9 days (95% CI: 7.8–7.9) in 1991 to 1994 to 3.5 days (95% CI: 3.5–3.5) in 2007 to 2010.^[24]^ The finding of decreasing trend in ALOS was similar to the results of other studies conducted in Taiwan.^[20,39–41]^ However, it is obvious that the ALOS of TKR in Taiwan was longer than that in United States, which suggests that there is still more room for improvement in TKR treatment.

The median charge of TKR in hospitals rose from $19,309 to $29,509 (1.53 times) over the period 1997 to 2004 in the United States.^[42]^ An obvious stability of TKR medical resource usage is observed in the last few years in the United States.^[24,43]^ A similar rise was observed in our study. The average hospital charges of TKR increased from $39.69 to $83.35 million (an increase of 110.00%). Many clinical parameters have been found to have a strong relationship with hospital charges in previous studies. Patients are inclined to look for hospitals with high surgical quantity and treatment from surgeons when the hospital charges increase.^[44,45]^ Another possible reason for the rise of the total medical expenditures is that population ages and life expectancy increases.^[46]^ Nevertheless, a lot of clinical parameters could not be examined in our study due to the collection of the limited data. First, some comorbidities may be under-reported by using ICD-9 codes. Second, it is not possible to gain detailed information related to lifestyle, exercise, diet, and other risk factors that may affect the TKR procedure from the insurance claims database. Third, we could not estimate real biologic factors, because they were not recorded in the NHIRD.

## 5 Conclusion

In conclusion, this study provides an analysis of TKR in Taiwan, and the results may be used as a reference for future planning of resources and budget for TKR in Taiwan. Further study may investigate the specific rehabilitation interventions and components following lower extremity joint replacement, so that we can better understand the effect of different treatment modalities.

## Author contributions

FHL wrote the manuscript. CCW, SLS participated in the design of the study, and helped to revise the manuscript. CCL participated in the design of the study. HCC, YLC, GSH participated in the design of the study, analyzed and interpreted the data. CL, HC carried out the statistical analysis. HLC, WS participated in the design of the study, and collection of data. HSL, SJY conceived of the study, and participated in its design and coordination and helped to draft the manuscript. All authors read and approved the final manuscript.

**Conceptualization:** Fu-Huang Lin, Hsiang-Cheng Chen.

**Data curation:** Fu-Huang Lin, Hsiang-Cheng Chen, Chin Lin, Yu-Lung Chiu, Herng-Sheng Lee, Hung Chang, Guo-Shu Huang, Wen Su, Chih-Chien Wang.

**Formal analysis:** Fu-Huang Lin, Hsiang-Cheng Chen, Chin Lin, Yu-Lung Chiu, Herng-Sheng Lee, Hung Chang, Guo-Shu Huang, Chih-Chien Wang.

**Investigation:** Hsueh-Lu Chang.

**Methodology:** Hung Chang, Hsueh-Lu Chang, Sui-Lung Su.

**Project administration:** Sui-Lung Su.

**Resources:** Shih-Jen Yeh, Sui-Lung Su.

**Software:** Shih-Jen Yeh, Sui-Lung Su.

**Supervision:** Sui-Lung Su.

**Validation:** Sui-Lung Su.

**Visualization:** Sui-Lung Su.

**Writing – original draft:** Fu-Huang Lin, Hsiang-Cheng Chen.

**Writing – review & editing:** Fu-Huang Lin, Hsiang-Cheng Chen.
